# Cardio-Pulmonary Parasitic Nematodes Affecting Cats in Europe: Unraveling the Past, Depicting the Present, and Predicting the Future

**DOI:** 10.3389/fvets.2014.00011

**Published:** 2014-10-09

**Authors:** Donato Traversa, Angela Di Cesare

**Affiliations:** ^1^Faculty of Veterinary Medicine, University of Teramo, Teramo, Italy

**Keywords:** *Aelurostrongylus abstrusus*, *Troglostrongylus brevior*, *Capillaria aerophila*, *Dirofilaria immitis*, cat, epidemiology

## Abstract

Various cardio-pulmonary parasitic nematodes infecting cats have recently been fascinating and stimulating the attention of the Academia, pharmaceutical companies, and veterinary practitioners. This is the case of the metastrongyloids: *Aelurostrongylus abstrusus* and *Troglostrongylus brevior*, the trichuroid: *Capillaria aerophila* (syn. *Eucoleus aerophilus*), and the filarioid: *Dirofilaria immitis*. Apparently, these parasites have been emerging in several European countries, thus, gaining an important role in feline parasitology and clinical practice. Under a practical standpoint, a sound knowledge of the biological, epidemiological, and clinical impact of cardio-respiratory parasitoses affecting cats, in addition to a potential risk of introduction, establishment, and spreading of “new” parasites in Europe is mandatory in order to understand the present and future impact for feline medicine and to address new strategies of control and treatment. The purpose of the present article is to review the current knowledge of heartworm and lungworm infections in cats, discussing and comparing past and present issues, and predicting possible future scenarios.

## Introduction

Parasitic nematodes affecting the cardio-pulmonary system of pets have recently attracted the attention of the Academia, pharmaceutical companies, and veterinary practitioners. This new interest has been stimulated by their apparent emergence in several European countries along with a re-discovered major role in clinical practice due to their pathogenic potential and the challenges posed in their diagnosis and control (1–4).

In Europe, the metastrongyloid *Aelurostrongylus abstrusus* and the trichuroid *Capillaria aerophila* (syn. *Eucoleus aerophilus*) are the most important nematodes affecting the respiratory system in cats. Metastrongyloid lungworms ranked within the genus *Troglostrongylus* have been considered affiliated to only wild felids for quite a long time, yet, recent records have shown the occurrence of *Troglostrongylus brevior* in domestic cats living in Europe, though only in Spain and Italy (5–10).

The filarioid *Dirofilaria immitis* is a nematode, which primarily infects the heart and pulmonary arteries of dogs and other canids, but it can infect cats as well. Nonetheless, its occurrence in cats is limited by the innate resistance of this host and infections are recorded, especially in those animals living in hyper-endemic regions, with a proportion of about 10–25% of the infected canine population (11, 12).

In the last decade, different drivers have changed the epidemiological scenario in Europe for these nematodes and, interestingly, there is evidence that various factors have presently been changing the distribution of cat cardio-pulmonary parasites in both endemic areas and previously free regions.

Indeed, climate changes, modifications in vector seasonal dynamics, movements in animal populations, shipping of goods, destruction of wildlife habitats, journeys of pets with their owners, may all play a role in the rise of cardio-pulmonary parasitoses (1, 4).

The majority of heartworms and lungworms infecting cats have an indirect life cycle, as they require an intermediate host for their development. Several species of mosquitoes, where *Culex pipiens pipiens* is the most important, transmit *D. immitis*, while a plethora of terrestrial mollusks act as intermediate hosts for *A. abstrusus* (13–16). Moreover, some species of gastropods may be competent vectors of *A. abstrusus* and *T. brevior* at the same time (17, 18).

The occurrence of cardio-respiratory nematodes is mainly influenced by the availability of competent gastropods and culicids in a given area, and at the same time the biology and the epidemiology of vectors and transmitted parasites is influenced by different factors, e.g., environmental temperature and humidity.

Contrariwise, *C. aerophila* develops directly in the environment, even though earthworms have been hypothesized to be facultative or paratenic intermediate hosts (13, 19). Drivers influencing the occurrence of lung capillariosis are still unknown, but modifications in wildlife habitats is likely at the basis of a common pattern of transmission between wild reservoirs and cats, with a possible emergence in domestic animals (20). Interestingly, the same factor could also influence the epidemiology of metastrongyloids and filaroids affecting cats (4, 8).

While changes in the current dispersion of cat parasites might be spurred by extrinsic and intrinsic factors, it should also be elucidated whether an increasing attention to these parasites has led to higher frequencies of documented diagnoses and/or it has partially or totally contributed to this apparent emergence.

The present review aims at discussing the past and present epidemiological scenarios for cardio-respiratory nematodes affecting cats, with a critical analysis of those drivers able to influence their occurrence in European territories, either singly or synergistically. Furthermore, new possible situations that could be expected in the near future, if the present trend continues, are discussed.

## The Past and the Present

### Climate changes

The development and survival of invertebrates transmitting nematodes is influenced by temperature, moisture, and water availability. Therefore, global warming is nowadays considered as a major factor potentially nurturing the dispersal and spread of pathogens transmitted by vectors (1, 21, 22). Importantly, climate changes are also likely to have an impact on the development and transmission patterns of cardio-pulmonary parasites.

Population dynamics and seasonal activities of mosquitoes are highly sensitive to temperature and moisture and, additionally, the development of *Dirofilaria* larvae in the insects is also known to be temperature-dependent. As a consequence, the transmission of *D. immitis* to vertebrates depends on a suitable climate allowing the survival and reproduction of mosquitoes and the larval development in the vectors themselves (23–25).

Indeed, recent literature on *D. immitis* has described a trend in its geographic spread, in both endemic and areas previously free of infection [reviewed in Ref. (4)]. First, the increase in average temperatures has actually influenced the abundance of mosquitoes and their seasonal survival in many territories of Europe, while, at the same time having an impact on the occurrence of filarial infections (2, 21, 26). Specifically, warmer temperatures may promote mosquito breeding and reduce their extrinsic incubation periods (25). Second, it is well known that environmental temperatures influence the development of larval *D. immitis* to the infective stage in the mosquitoes. The incubation of *D. immitis* larval stages from microfilariae to infective L3 in mosquitoes is influenced by temperature thresholds. At 30°C, the development is completed in 8–9 days (e.g., in *Aedes vexans, Aedes triseriatus, Aedes trivittatus*, and *Anopheles quadrimaculatus*) increasing to 10–14 days at 26°C, 17 days at 22°C, and 29 days at 18°C (23).

Different forecast models, using wide or local scale temperature data, have been developed to predict the occurrence and seasonality of *Dirofilaria* (2, 21, 26–29). These climate-based systems usually employ the concept of growing degree days (GDD), i.e., 18 days are necessary for the development of *Dirofilaria* infective larvae when the average temperature for the day is 1°C above the threshold temperature. These models were based on the following evidence: development of *Dirofilaria* do not proceed below a threshold of 14°C (23), a requirement of 130 GDD for the larvae to reach infectivity and the maximum life expectancy is 30 days for a vector mosquito (27, 28). Some years ago these models showed that due to the rise in average temperatures and climate changes, most of the European countries have become suitable for *Dirofilaria* transmission, with a lengthening in the duration of the filarial transmission season (26, 29). After <10 years, the most recent information demonstrates the establishment of new foci of dirofilariosis in pets and wildlife in Europe and an increased prevalence in regions that previously experienced only sporadic infections (3, 4). Indeed, the epidemiological profile of dirofilariosis has substantially changed over the last decade. Until 2001, cardio-pulmonary dirofilariosis was mainly found in southern European countries (e.g., Italy, Spain, and France), which are considered historically endemic/hyper-endemic countries (3). An expansion of *D. immitis* toward central and northern Europe (e.g., Russia, Hungary, Czech Republic, Slovakia, Croatia, Serbia) has been reported in the last years (3, 4). For instance, autochthonous cases of *D. immitis* infection in dogs were reported in Hungary in 2009 (30), in Slovakia in 2010 (31), and in Poland in 2012 (32) and, in 2014, *D. immitis* was detected for the first time in *C. pipiens/torrentium* mosquitoes in Germany (33). Additionally, veterinarians have recently reported different cases of cardio-pulmonary dirofilariosis in Germany (34).

**Figure 1:**
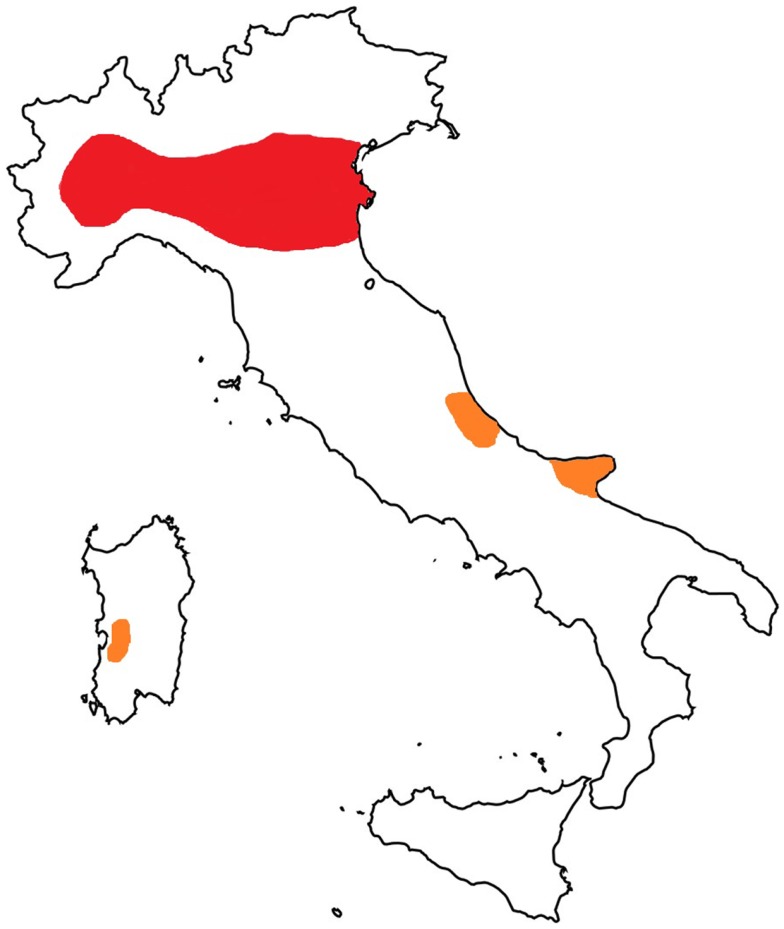
**Map of Italy: hyper-endemic area for *Dirofilaria immitis* (in red) and new autochthonous foci of infection in cats (in orange)**.

A prevalence of microfilariaemic hosts (mainly dogs and foxes) and the presence of competent vectors raise the rate of infection in the mosquito population, which is directly related to the risk of animals and humans being infected. *D. immitis* infections in cats in Europe have been reported in Italy, France, and Portugal, with an increasing frequency in areas where the disease is endemic in dogs [reviewed in Ref. (35)]. As a key example, in Italy, the infection in cats has been traditionally diagnosed for a long time in northern Italy with a prevalence that goes from 9 to 27% in the hyper-endemic area of the Po River valley (36). In a recent study carried out in northern Italy, the prevalence of feline heartworm disease has been estimated as 10% of the known prevalence of the infection in dogs (12). Interestingly, autochthonous cases have recently been described in cats in the central (37) and southern regions of Italy (38) (Figure 1). On the other hand, it should be taken into account that the development of *D. immitis* in cats takes longer than in dogs, cats are generally amicrofilariaemic and clinical signs are generally transient, sometimes with sudden death (11, 12, 35, 39). Therefore, given that the diagnosis of *D. immitis* in cats is highly problematical, the infection is likely to be underestimated in feline patients from several territories where the parasite is endemic.

**Figure 2:**
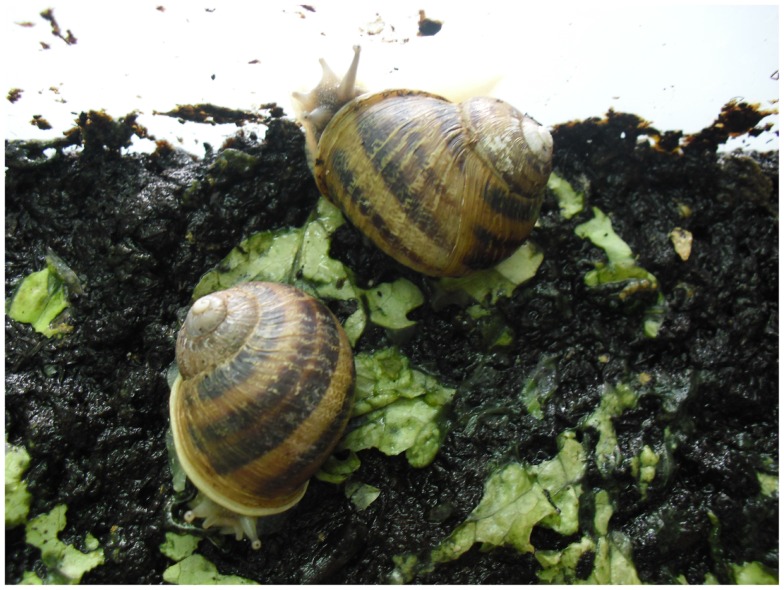
***Helix aspersa* snails**.

The life cycle and the dynamics and activity of the population of gastropods are also sensitive to temperature. At the same time, the development of nematode larvae in snails and slugs is known to be influenced by temperature changes. The Mediterranean *Helix aspersa* (Figure 2) edible snail is one of the most widely spread land snails in the world (40). Deliberately or accidentally imported, this species have recently become a pest outside its native Mediterranean range (41). Environmental temperatures may influence the biological cycle of the cat lungworm *A. abstrusus* and, in particular, the higher the average temperature the higher the rate of larval development in *H. aspersa* (15). In addition, larvae of *A. abstrusus* (and of *T. brevior*) may survive in overwintering *H. aspersa* (18). Given that the rise in temperatures has the potential to nurture the development of *A. abstrusus*, it is plausible that climate changes have contributed (and are contributing) to the apparent spreading of feline metastrongyloids in Europe. In fact, *A. abstrusus* has traditionally been considered sporadic in Europe but, recently, the parasite is constantly and increasingly reported in different areas of the continent (Table 1) suggesting that a possible expansion in its geographical range and supporting a rise of prevalence in cats, with a rate of up to 25–50% (1, 37, 42–46).

**Table 1:** **Reported prevalence or clinical cases (S) of cat aelurostrongylosis in different European countries**.

Country	*Aelurostrongylus abstrusus* (%)	Reference
Albania	39.7–50	(44, 45)
Belgium	S	(1)
Croatia	0.38–22	(1)
Denmark	S	(1)
France	S	(1)
Germany	0.5–15.3	(47, 48)
Great Britain	3.6–10.6	(49, 50)
Greece	S	(1)
Holland	2.6	(1)
Hungary	14.5	(51)
Ireland	S	(1)
Italy	1.8–24.4	(1, 43, 52)
Norway	S	(1)
Poland	S	(1)
Portugal	17.4	(42)
Romania	5.6	(1)
Spain	1	(1)
Turkey	S	(1)

Parasitic nematodes belonging to the genus *Troglostrongylus* have been regarded to infect only wild felids (8, 17, 53). Nonetheless, the finding of *T. brevior* and *Troglostrongylus subcrenatus* in kittens on the islands of Ibiza and Sicily (5, 6) has renewed the scientific interest on these metastrongyloids (8). *Troglostrongylus brevior* was described for the first time by Gerichter (17) and, since then, no other peer-reviewed international article has recorded the infection in domestic hosts. There is only a local report from Italy, which reported its occurrence in a wildcat and in a cat defined as “feral” in central Italy (54).

In the past few years, *T. brevior* has been recorded in other areas of Italy, i.e., in Sardinia Island and in southern and central continental territories (7, 9, 10, 46, 55, 56). The reasons for this present rise of descriptions are unknown. Given that different species of mollusks may transmit *A. abstrusus* and *T. brevior* (17, 18), changes in vector phenology nurtured by global warming might have a role in the present rise of cases for these cat lungworms. With specific regard to *T. brevior*, modifications in the life of wild reservoirs could be another cause of this apparent emergence (see below).

**Figure 3:**
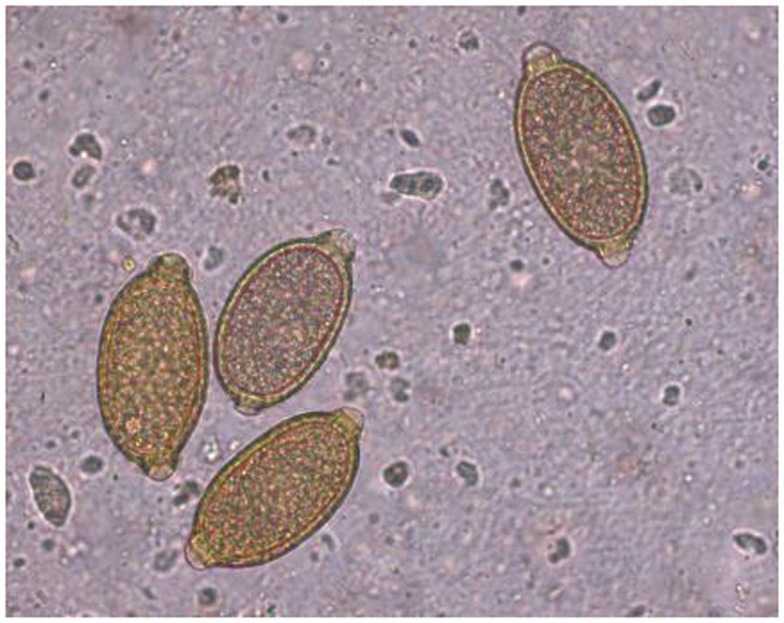
***Capillaria aerophila* eggs**.

Feline infection by *C. aerophila* has often been considered sporadic and sub-clinical. However, the parasite has recently been described in cats in both clinical cases and during copromicroscopy-based surveys (9, 10, 52, 57–59), along with zoonotic infections in humans (60). The presence of *C. aerophila* is guaranteed by the high resistance of its eggs (Figure 3) even in harsh environmental conditions and by the ubiquity of earthworms, pending they actually intervene in the biological cycle of this worm. No thorough information is available on the impact of different temperature ranges in the maturation of *C. aerophila* eggs in the soil. However, a study has shown that eggs released by infected hosts start to develop after 35 days and mobile larvae are observed in the eggs after 2 months at 20 ± 1°C and 80–85% of temperature and relative humidity (61). Further studies are necessary to understand whether different temperature values may change the speed and the rate of egg maturation on the environment. Therefore, at the moment it can only be argued that climate changes might partially influence the epidemiology of lung capillariosis, and that the occurrence (and spreading) of *C. aerophila* in cats may be due to other drivers (see below).

### Vectors

In the past decades, vectors and parasites have been introduced into European areas due to human intervention (e.g., urbanization), movements of people across countries, changes in the habitats of animals, the legal and illegal trade of animals and goods (4, 62).

The capability of commercial trade and movement of goods in favoring the introduction of competent vectors and the establishment of parasites from endemic regions to free areas has been demonstrated by the recent expansion of *D. immitis* (and *Dirofilaria repens*) in Europe. In fact, this phenomenon has somehow matched the introduction and spread of the Asian tiger mosquito *Aedes albopictus* (4). As a key example, the rapid distribution of *A. albopictus* throughout Italy has likely broadened the dirofilariosis range to previously free southern regions. Indeed, this mosquito may extend the animal (and human) risk of exposure to *D. immitis* for the day long and during the whole year in southern areas, especially in urban habitats. On the basis of retrospective evidence, it is clear that the expansion of dirofilariosis in Italy and Europe has followed the introduction and the spreading of *A. albopictus* in the continent by 1990 from Italy via commercial trades (4, 63).

In Italy, *D. immitis* and *D. repens* were found in natural populations of *A. albopictus* in 2000–2002 (64), and the rapid spread of this mosquito throughout the country has likely broadened the geographic range of dirofilariosis to southern regions not previously infected, despite *C. p. pipiens* had already established nationwide (4, 65). The geographic sympatrical occurrence in different European countries of *A. albopictus* and *C. p. pipiens* has become of great epidemiological relevance. Both mosquitoes have diurnal and nocturnal biting activities and may enhance the risk of infection to animals and humans in endemic areas throughout the day. In addition, *C. p. pipiens* has recently changed its endophagic and anthropophagic behavior in Central and North Europe, where it currently bites outdoors, as it was the case for southern parts of the continent (4, 66). The co-presence of both vectors could have promoted the risk of infection in central and southern regions of Italy (4) where new foci of dirofilariosis have been reported (37, 38). Interestingly, this pattern also overlaps with the spread of *Dirofilaria* spp. in central and north-eastern Europe (e.g., Switzerland, Czech Republic, Hungary, Serbia, and Slovak Republic) (2, 4, 26, 67–69).

Such changes might indeed have implications in the occurrence of *D. immitis* in cats. In fact, a study carried out in an *D. immitis*-endemic area, aiming at understanding the attraction of mosquitoes to domestic cats, has shown that *Culex* species were those most frequently found with feline blood meal, followed by *Aedes* species that also fed on feline blood. While *Culex quinquefasciatus* is mostly associated with cats infections (39, 70), also *A. albopictus* (and other closely related species) feed on feline blood and therefore they might be indeed involved in the transmission of *D. immitis* (71).

Analogously, the geographic spread of slugs and snails driven by conditions of global warming might play a role in the incidence and distribution of gastropod-transmitted parasites (72–75). Among the different species of mollusks that transmit cat metastrongyloids, *H. aspersa* plays a major role in the biological cycle of lungworms. This land snail is now abundant in all anthropized areas of those regions with a climate suitable for its development (76) and, besides intentional introductions into previously free areas for farming purposes, it has also been accidentally introduced by the movement of plants and vegetables (41). The subsequently rapid adaptation of this snail to novel environmental conditions has partially been attributed to their ability in changing some of their life-history traits, such an increase in reproductive investment involving earlier maturity at a bigger size and a shorter generation time could thus explain its invasive success (41, 77). At the moment, *H. aspersa* is among the most common land snails in the world and it is also extensively farmed for human consumption in several countries (40). These snails are usually farmed in outdoor pens, which indeed may increase the risk for the biological interaction between snails, lungworms, and suitable felid hosts. Recently, infective *A. abstrusus* larvae have been found in the slug *Arion lusitanicus* in Poland (16). It is worth mentioning that *A. lusitanicus* is widespread and since the 1950s it has become established in many European countries, where it is now considered as a serious pest in agriculture and private gardens, parks, and forests (78). Indeed, *A. lusitanicus* has a high degree of plasticity in its thermal biology, it spreads rapidly in new geographical areas to which it has migrated and tends to occur in very high densities (79). The finding of *A. abstrusus* in this slug could represent a new potential source of infection for cats (16).

### Wildlife

Current changes in phenology and biology of wildlife is a key driver in changing the epidemiology of internal and external parasites infecting companion animals, and this is also true for cardio-pulmonary nematodes. In fact, wild animals are suitable reservoirs of poorly species-specific parasites which, in this particular case, may infect cats as well.

Destruction and/or reduction of natural habitats and conurbation have recently induced feral and wild felids and canids, especially foxes, or even mustelids, to move into new hospitable environments and to look for anthropogenic food sources, e.g., the suburbs and cities. As a consequence, these movements favor the spread of wildlife parasites and increase the contact between parasites harbored in wildlife and pets (1, 80–82).

**Figure 4:**
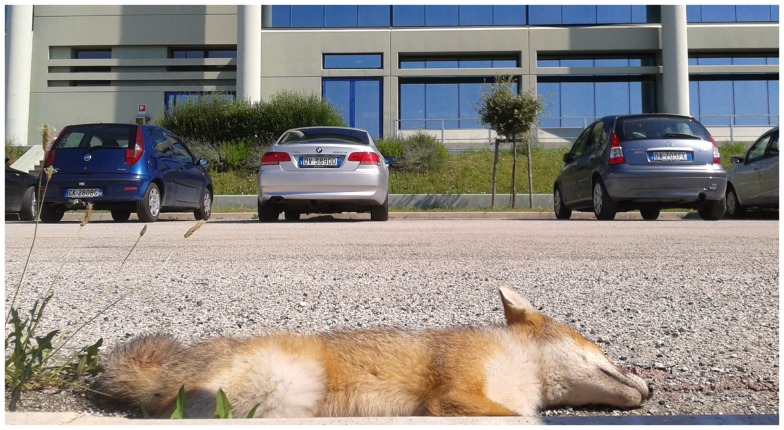
**Dead red fox in a peri-urban area of the municipality of Teramo, Abruzzo Region, Italy**.

The presence of red foxes in cities and peri-urban areas (Figure 4) has been repeatedly evocated in concurring to the emergence of heartworms and lungworms in domestic hosts. Remarkably, red foxes are common reservoirs for *D. immitis* in European countries. Specifically, red foxes infected by *D. immitis* are present in Italy, Spain, Bulgaria, and Hungary, with a prevalent rate up to 32% (35, 83–85). In accordance with the climate-based forecast prediction model, the prevalence and intensity of heartworm infection in wild canids in Hungary, which was not considered an endemic country until 2007, is similar to that observed in Mediterranean areas of Europe (85). Foxes infected by *D. immitis* in Hungary were not microfilariaemic, and no L1s were found in the uterus of the female worms recovered from necropsy, which were very few. The presence of a low number of adult nematodes without microfilariaemia could suggest that in some epidemiological settings, foxes are not adequate reservoirs for *D. immitis* (85, 86). However, microfilariemic foxes are present in Italy (84) and, outside Europe, in Australia (87), indicating that they are reservoirs of this nematode (4). As wild carnivores could be sentinels for the spread of *D. immitis*, the impact of foxes on the transmission dynamics of dirofilariosis and the reason of the different presence of microfilariae and adult worms in these hosts should be further investigated.

While red foxes could be a wild reservoir of the parasite in different epidemiological scenarios, other wild hosts found infected with *D. immitis* (e.g., wolves, jackals, otters, and ferrets), are likely to represent an epi-phenomenon for this infection (4).

The recent findings of *T. brevior* in domestic hosts are of difficult explanation. At the moment, it is hard to understand whether *T. brevior* (i) infects domestic cats in confined areas and/or under certain epidemiological settings; (ii) has been misdiagnosed as the more common *A. abstrusus* for a long time; (iii) is currently emerging in previously free areas and/or hosts due to a combination of climatic and biological factors. If one bears in mind that nematodes of *Troglostrongylus* genus have been traditionally considered proper of wild species of felids, the role of wildlife in causing the present cases of infections in domestic cats may possibly be attributed to wild reservoirs. Indeed, various information suggest that the occurrence of *T. brevior* in domestic cats could be due to a possible affiliation with this host mainly in certain regions or under conditions, which are particularly favorable for bridging infections between wild and domestic animals. It is possible that *T. brevior* could usually be confined in wild or feral hosts but able to change its usual affiliation under suitable epidemiological factors and routes of development and transmission that, at the same time, may spur the dispersion of *A. abstrusus* (8, Di Cesare et al., submitted).

Considering that the proper hosts of *T. brevior* are likely wild felids, the limited number of these animals even in their natural habitats might account for the marginal occurrence of this lungworm. Indeed, apart from the record from Ibiza Island, all reports of *T. brevior* are from Italy, where three subspecies of *Felis* spp. are present, i.e., the Sardinian wildcat *Felis silvestris libyca*, the European wildcat *Felis silvestris silvestris*, and the domestic cat *Felis silvestris catus*. These three *Felis* subspecies have a negligible level of hybridization, but recent demographic and ecological conditions have led to a certain degree of cross-breeding between wild and domestic cats (88). Moreover, some territories of Italy have faced an expansion in the geographical distribution of *F. s. silvestris* (89). It can be argued that some ecological and epidemiological drivers might recently have contributed in a spill-over of *T. brevior* from wild to domestic felids, in particular niches and under certain routes of transmission. This seems to be supported by the fact that *T. brevior* has been found exclusively where populations of *F. s. silvestris* and *F. s. libyca* may have the potential to come into contact with domestic cats. A recent retrospective work from endemic areas of Italy has evaluated the presence of lungworms in domestic cats, which received a diagnosis of respiratory parasitosis or with compatible lung lesions (Di Cesare et al., submitted). This study demonstrated that *A. abstrusus* was the major lungworm implicated in cat respiratory parasitosis in these regions of Italy in 2002–2013 and that *T. brevior* was present with low-infection rates (Di Cesare et al., submitted).

Recent data on the helminthofauna of *F. s. silvestris* in Italy showed that *T. brevior* is spread in wildcat populations of northern and southern Italy (90, 91). In particular, a high-infection rate (71.4%) by *T. brevior* has been detected in wildcats from areas where this parasite has been described in some cats (6, 7, 55).This evidence indicates that the infection is widespread among wild cats where appropriate ecological niches occur and that they could act as reservoir for this parasite (91). On the other hand, the high-infection rate in wild cats, in spite of occasional reports of troglostrongylosis in domestic cats from the same areas, indicates that *F. s. silvestris* is the natural host of *T. brevior* and may act as a spreader of the parasite. Indeed, the reduction of natural habitats may force wild and domestic cat to occupy the same habitats (92) and, as a consequence, a spill-over of *T. brevior* might occur from wild reservoirs to domestic hosts.

*Capillaria aerophila* infects a broad spectrum of animals, thus, lung capillariosis may occur in different species of wild and domestic carnivores. Although the infection in cats has often been considered sporadic, in the past decade, *C. aerophila* has been recorded in different countries (47, 51, 52, 57, 59, 93, 94). It has recently been shown that distinct genetic populations of *C. aerophila* are shared between foxes, beech marten, cats, and dogs in European countries, supporting the existence of common patterns of transmission between wildlife and pets (20). Interestingly, 15 different haplotypes were characterized and 5 were shared between pets in Italy and wildlife in Europe with 3 genetic sub-populations infecting cats and foxes in Serbia, Romania, and Portugal. The existence of haplotypes shared between cats and wildlife in different countries suggests that common patterns of transmission for cardio-pulmonary nematodes could be of high epidemiological importance (20). It should also be borne in mind that *C. aerophila* may infect humans and that the most recent published case of human lung capillariosis has been reported in Serbia, where the infection rate of lung capillariosis in foxes is very high (60, 95). Importantly, the genetic haplotypes of *C. aerophila* found in Serbian foxes have also been described in cats (and dogs) in Italy and in foxes in Romania (20). Nonetheless, a comprehensive knowledge of the epidemiology of *C. aerophila* (e.g., range of hosts and geographic distribution) is still poor and it is difficult to assess to what degree this parasite may be spreading or what influence different factors may have on the current distribution of lung capillariosis.

## What to Expect in the Near Future?

An increased awareness is presently spurring new studies on felid heartworms and lungworms, and this could explain the rise of records of the infections they cause. These parasites have been regarded as occasional in cats for a long time, thus, lack of awareness and/or misdiagnosis could have resulted in an underestimation in the past. Indeed, *A. abstrusus* and *C. aerophila* may induce both sub-clinical and clinical infections, with varying clinical outcomes depending on different variables such as worm burden, age, and immune response of the infected animal, and concomitant diseases. Clinical signs, which are more severe in young, debilitated, and/or immunosuppressed animals, are common to many other respiratory diseases in cats (e.g., fungal, bacterial, and neoplastic diseases). Hence, cases of infection may be easily missed if appropriate laboratory methods are not applied by skillful operators (8, 96). For instance, although parasitological methods are able to detect *A. abstrusus* L1 and *C. aerophila* eggs, they are generally not performed by vet practitioners and suffer of diagnostic shortcomings, e.g., intermittent shedding of larvae and eggs (1).

### Cardio-pulmonary filariosis

Cardio-pulmonary filariosis in cats may be characterized by a prolonged prodromal period with no apparent clinical signs, evolving in the sudden death of the animal (97, 98). When present, clinical signs are aspecific and the conventional diagnostic methods are unreliable to diagnose the disease in cats (98). In fact, the detection of circulating larvae in the bloodstream of cats is unlikely due to practically null and/or short-lasting periods of microfilariemia and to non-patent infections (11, 98). In the near future, ecological and biological drivers might indeed nurture the dispersion of *D. immitis* in cats, thus, veterinarians should be vigilant on possibly emerging feline dirofilariosis and improve their knowledge and awareness on diagnostic, control, and treatment methods.

### Lungworms

While adult animals harboring *T. brevior* are subclinically infected, clinical signs are severe in young animals and the infection is often fatal in kittens (5–10, 55, 56, 99). Indeed, the clinical severity of troglostrongylosis casts shadows on the hypothesis that *T. brevior* has been misdiagnosed in cats for a long time with the more common *A. abstrusus*. In fact, descriptions of severe or fatal aelurostrongylosis in literature are unusual, while the high level of pathogenicity of *T. brevior*, especially in kittens, makes it unlikely that these fatal cases in previous years were missed. Records of fatal infestations would have been more numerous if troglostrongylosis had so often been mistaken for aelurostrongylosis, and this is not the case (8). Therefore, it is plausible that some drivers are someway spurring the dispersion of the parasite in domestic hosts living in certain areas. Thus, if troglostrongylosis is a truly emerging parasitosis of cats, it could become an important threat in feline clinical practice in the near future.

These considerations mirror the recent scientific analysis carried out to explain the occurrence and the emergence of the high-pathogenic mollusk-borne nematode *Angiostrongylus vasorum* in dogs (1, 37, 82, 100, 101). With similar life cycle patterns, the same drivers involved in the spread of *A. vasorum* would likely also have an effect on *A. abstrusus*, and possibly *T. brevior*. In fact, it can be argued that ecological factors are nurturing *T. brevior* infection in cats, at least in some regions, and that if these factors continue influencing the epidemiology of the disease, the nematode could expand its distribution in territories larger than the islands, southern and central areas of Italy (8). For instance, apart from climate change and the spreading of mollusks, another factor potentially promoting the emergence of cat troglostrongylosis is the possible spill-over of the nematode in those areas where populations of wild cats are living, i.e., the same regions where *T. brevior* has so far been recorded in domestic hosts (91, Di Cesare et al., submitted).

The infection by *C. aerophila* in cats is not commonly included in differential diagnosis in current practice and should be considered a neglected disease (1). Nevertheless, the clinical impact on feline patients, the increased awareness of its importance in veterinary practice, and the recent evidence of a spreading of this pathogen likely promoted by wild reservoirs would likely cause a rise of documented diagnosis in the near future. Veterinarians should become vigilant on this infection also because the parasite may infect different species of pets, and humans as well.

## Concluding Remarks

In conclusion, evidence supports a present emergence of *D. immitis, A. abstrusus*, and *C. aerophila*, while several questions remain to be answered for *T. brevior*.

The further increment in temperatures expected in the future has the potential to expand areas of mosquito colonization, to favor the invasion of new areas and, possibly, to enhance their vectorial capability. Remarkably, a northward spread of the geographic occurrence of *A. albopictus* in western and central Europe up to the middle of the century has been assumed due to the general trend of increasing climatic suitability in regions that nowadays are unfavorable for this insect (102). This would also influence the rate of *D. immitis* infection in animals and people, the lengthening in the seasonal activity and the consequent rise in the biting rates received by susceptible hosts living in both traditional and new endemic areas. Although various mosquitoes (including *C. p. pipiens*) are more attracted to dogs than to cats (39, 70), the increase in density and their high adaptability in several territories, along with the presence of domestic (e.g., dogs) and wild (e.g., foxes) microfilariemic reservoirs, could increase the ability of *D. immitis* to establish transmission cycles and to also maintain the infection in feline populations.

With the present rise in temperatures, it can also be argued that the overwintering period of terrestrial mollusks will be shortened and that their seasonal activity will become longer. Hence, in the near future, cats with outdoor access would be more prone to be infected with lungworms.

Other than climatic variables, it should be elucidated on how and if other factors (e.g., wildlife movements, pet journeys) will further influence the current spread of these nematodes.

It is important to note that the lack of specificity of clinical signs of infections caused by cardio-pulmonary nematodes and the difficulties in the differential, clinical, and laboratory diagnosis may lead to misdiagnosis and/or missed diagnosis. Hence, animals have the potential to remain infected and untreated and, when they travel with their owners, various parasites may be transported from endemic to free regions.

If the emergence of these parasites is not only apparent but also a matter of fact, and if their dispersion continues to follow current trends, in the near future we could be faced with an increase of prevalence of feline heartworm disease and of lung capillariosis and aelurostrongylosis, along with cases of “new” infections, i.e., troglostrongylosis.

Under a practical standpoint, these potential changes would pose major clinical challenges in feline clinical practice. Veterinarians, parasitologists, and pet owners should be aware of the existence of cardio-parasitoses in cats and of the potential risk of introduction, establishment, and spreading of various nematodes, which might shortly alter the scenario of feline parasitology and clinical practice in a manner not easily foreseeable. This is particularly true in geographical regions considered free of infection and that has become or will become infected with “new parasites.”

## Author Contributions

Donato Traversa conceived, drafted, and revised the manuscript. Angela Di Cesare contributed to conceiving and drafting the manuscript, and prepared sources of literature, figures, and tables. Both authors have approved the version to be published and agree to be accountable for all aspects of the work in ensuring that questions related to the accuracy or integrity of any part of the work are appropriately investigated and resolved.

## Conflict of Interest Statement

The authors declare that the research was conducted in the absence of any commercial or financial relationships that could be construed as a potential conflict of interest.
